# Hydroxyapatite (HA) coating appears to be of benefit for implant durability of tibial components in primary total knee arthroplasty: A systematic review of the literature and meta-analysis of 14 trials and 926 evaluable total knee arthroplasties

**DOI:** 10.3109/17453674.2011.590762

**Published:** 2011-09-02

**Authors:** Jeffrey D Voigt, Michael Mosier

**Affiliations:** 99 Glenwood Rd., Ridgewood, NJ; Washburn University, Topeka, KS, USA; Correspondence JDV: meddevconsultant@aol.com

## Abstract

**Background:**

It is unclear whether there is a clinical benefit to adding hydroxyapatite (HA) coatings to total knee implants, especially with the tibial component, where failure of the implant more often occurs. A systematic review of the literature was undertaken to identify all prospective randomized trials for determining whether the overall clinical results (as a function of durability, function, and adverse events) favored HA-coated tibial components.

**Methods:**

A comprehensive literature search was performed for the years 1990 to September 16, 2010. We restricted our search to randomized controlled trials involving participants receiving either an HA-coated tibia or other forms of tibial fixation. The primary outcome measures evaluated were durability, function, and acute adverse events.

**Results:**

Data from 926 evaluable primary total knee implants in 14 studies were analyzed. Using an RSA definition for durability, HA-coated tibial components (porous or press-fit) without screw fixation were less likely to be unstable at 2 years than porous and cemented metal-backed tibial components (RR = 0.58, 95% CI: 0.34–0.98; p = 0.04, I^2^ = 39%, M-H random effects model). There was no significant difference in durability, as measured from revision and evaluated at 2 and 8–10 years, between groups. Also, functional status using different validated measures showed no significant difference at 2 and 5 years, no matter what measure was used. Lastly, there was no significant difference in adverse events. Limitations included small numbers of evaluable patients (≤ 50) in 7 of the 14 trials identified, and a lack of “hard” evidence of durability with need for replacement (i.e. frank failure, pain, or loss of functionality).

**Interpretation:**

In patients > 65 years of age, an HA-coated tibial implant may provide better durability than other forms of tibial fixation. Larger trials should be undertaken comparing the long-term durability, function, and adverse events of HA-coated implants with those of other porous-coated tibial implants in younger, more active OA patients.

## Rationale

Experts have recommended that uncemented TKA is preferable in patients < 60 years of age (Dorr 2002), for a number of reasons. Younger, more active patients can place higher stresses on implants and on their fixation to the bone. These higher stresses can result in osteolysis and loosening due to wear debris (including cement wear debris) generated over many years (Hoffmann et al. 2002). Considering that TKA implants last 10–15 years, the need for revision surgery and preservation of bone is of importance for younger patients. Uncemented implants may afford this.

The price of implants remains a major cost consideration for these types of procedures in the US. Hydroxyapatite (HA) coating on implants is usually an added cost (to an already expensive item) for providers, insurers, and patients. HA coatings may be applied to non-porous or porous surfaces. HA coatings are osteoconductive, meaning that they facilitate the formation of bone structure and may aid in more stable fixation over the years. Preclinical studies have shown that HA coatings can enhance the stability of implants by promoting early bone ingrowth even in the presence of interfacial gaps or initial interfacial instability (Dumbleton et al. 2004). HA-coated knee components have shown less radiolucency, with bone-filling gaps around the implant (Epinette et al. 2004). Furthermore, up to 12-year follow-up of HA coated, cementless total knee replacements in a cohort of young, active patients has demonstrated excellent results (Tai and Cross 2006). Based on early findings, there may be clinical benefit to adding HA coatings to total knee implants, and most especially to tibial components, where failure of the implant more commonly occurs. However, no one has yet examined the highest-quality evidence in its entirety for these types of implants (i.e. evidence from prospective randomized trials).

## Objectives

We examined all the published prospective, randomized clinical trials (level-1 evidence), comparing patients with OA who received HA-coated tibial components or other tibial designs including uncemented porous coated metal components, uncemented press-fit metal components, and cemented metal components (porous and non-porous coated) to determine whether the clinical results of durability, function, and adverse events (both short and longer term) seen with HA-coated tibial components were superior.

The following hypotheses were tested: 1. HA-coated tibial components as part of a total knee implant system have better durability than metal-backed (MB) tibial components (porous-coated uncemented, press-fit uncemented, or cemented) irrespective of the manufacturer/brand and patient age at time of implant over the short term (2 years), and longer term (≥ 8 years). 2. HA-coated tibial components as part of a total knee implant system have better functionality than MB tibial components (porous-coated uncemented, press-fit uncemented, or metal-backed cemented) irrespective of the manufacturer/brand and patient age at time of implant over the short term (2 years) and longer term (≥ 8 years). 3. HA-coated tibial components as part of a total knee implant system have a lower incidence of adverse events/complications than MB tibial components irrespective of the manufacturer/brand or method of fixation (e.g. cemented, porous coated).

## Methods

The Cochrane Review Manager software version 5.0.2 was used in assembling and reviewing the data (Cochrane 2008).

### Protocol

A study protocol was not published prior to this analysis. However, the above hypotheses were developed a priori, i.e. before the results were available. These hypotheses were developed based on a previous current concept review (Dumbleton et al. 2004) and on studies that have been published (not level-1 evidence) on the positive effect of HA coating on implant durability and function (Geekink et al. 1988, Rahbek et al. 2000, Dorr 2002, Dumbleton et al. 2004, Akizuki et al. 2003, Kane et al. 2003, Epinette et al. 2004, Rahbek et al. 2005, Tai and Cross 2006).

### Eligibility criteria for considering studies for this review

*Types of studies.* Randomized controlled trials (RCTs) were eligible for inclusion if they compared the use of an HA-coated tibial component and any other metal-backed tibial component in the short and longer term, specifically examining the durability (length of time the implant survived in the patient without having to perform a repeat procedure for loosening or pain or loss of function—i.e. revision surgery), functionality and pain (using validated scoring systems such as the Knee Society score, HHS, WOMAC (SF-36), LEFS), and adverse events.

Durability and/or functionality (as defined below) must have been one of the primary outcome measures evaluated in the trial.

*Definitions used for eligibility criteria.* The following definitions were used to determine clinical outcomes: durability, function, and adverse events. The definitions below are consistent with other technology assessments in total knee replacement (Kane et al. 2003) and with the widely used maximum total point motion analysis as defined by Ryd et al. (1995).

*Durability* was defined as the quality of fixation of the tibial component using radiographic analysis, or the length of time a total knee implant survives in a patient without having to replace it due to loosening/instability, wear, pain, or loss of functionality. Longitudinal radiographic analysis has been used increasingly over time as an accurate tool for assessment of micromotion in orthopedic implants, and is highly predictive of clinical loosening and revision (Ryd et al. (1995), especially when measured relatively early in the life of the implant (after 1–2 years). It has been found that continuous component migration represents defective fixation that shows very early. A maximum total point motion (MTPM) of > 0.2 mm/2 years implies that revision due to loosening can be predicted. (MTPM is a 3-dimensional vector, with the vectors being: (1) the x plane, which corresponds to medial migration of the component; (2) the y plane, which corresponds to proximal migration; and (3) the z plane, which corresponds to posterior migration). Thus, length of time in years that a tibial implant survives without having to replace it, or MPTM > 0.2 mm/2 years was used to define durability (indicating implant would likely fail).

*Functionality* was defined as the ability of the recipient of a total knee implant to function (including toleration of pain) in everyday living, as measured by a number of validated scoring systems including: the Knee Society score, Hospital for Special Surgery score (HSS) (Insall et al. 1976), the Western Ontario and McMaster University arthritis index (WOMAC), the SF-36/SF-12 health survey, the Oxford knee score, the EuroQol-5D, and the lower extremity functional scale (LEFS). In all measures, the higher the score, the better the patient is regarding clinical and functional outcome. The Knee Society score (KSS) is a tool for assessing a patient's: pain, flexion (range of motion), and knee alignment and function (walking ability, stair climbing ability, and need for walking aids). It is a surgeon-assessed, variable weighted score which results in a “clinical” score for the patient. The HSS score is also a surgeon's assessment of a patient's pain, range of motion/flexion, function, knee alignment, stability, and need for walking aids. The WOMAC index (a patient-assessed index) is used to assess patients with osteoarthritis of the knee using 24 parameters, and it focuses on pain, stiffness, and social and emotional function. The SF-36 health survey (patient-assessed) yields a profile of functional health and well-being scores as well as a psychometrically-based health utility index. It is a general measure and does not target any specific age, disease, or treatment group. A shorter version of the SF-36, the SF-12 form, is also used for functionality. The Oxford knee score (OKS) is a patient-assessed equally weighted score. The OKS assesses pain and function from the patient's point of view. The higher the score, the more “functional” a patient is. The EuroQol-5D is a standardized instrument for use as a measure of health outcome. It is designed to assess a wide variety of health conditions and treatments, and provides a simple descriptive profile and a single index value for health status. It is designed for self-completion. EuroQol-5D consists of 5 dimensions: mobility, self-care, usual activity, pain/discomfort, and anxiety/depression. The LEFS (also developed at MacMaster University) measures the functional effectiveness of an intervention over time (also patient-assessed).

*Adverse events* were defined as complications resulting from the surgical procedure and/or subsequent need for surgical intervention and included: DVT, instability due to late varus or valgus with need for surgical intervention, patellofemoral problems with need for surgical intervention, knee flexion problems (including stiffness) with need for surgical manipulation, hematoma requiring evacuation, allergic reactions to medications and infections (over the life of the implant).

*Types of participants.* Any person undergoing a first-time total knee arthroplasty.

*Interventions:* Patient of any age undergoing a total knee implant that contained an HA-coated tibial component vs. other total knee implant with a different mode of fixation of the tibial component. Comparison groups in prospective randomized trials included:

- Total knee implant with HA-coated tibial component (with or without porous coated tibial undersurface; and with or without screw fixation) compared to a total knee implant with porous coated or press-fit stemmed metal tibial tray with polyethylene bearing surface.
- Total knee implant with HA-coated tibial component (with or without porous coated tibial undersurface; and with or without screw fixation) compared to a total knee implant with porous coated modular (for polyethylene insert) metal tibial tray with polyethylene bearing surface; with or without screw fixation.
- Total knee implant with HA-coated tibial component (with or without porous coated tibial undersurface; and with or without screw fixation) compared to a total knee implant with a cemented metal tibial tray with polyethylene insert bearing surface.

*Types of outcome measures*

- Durability of implant with at least 2-year follow-up (including availability of MTPM or radiographs at 2 years).
- Functionality of implant with at least 2-year follow-up.
- Adverse events occurring during procedure or afterwards, such as deep vein thrombosis, allergic reactions, infections, decreased range of motion.

*Secondary outcomes*

- Osteoarthritis vs. rheumatoid arthritis patients (HA vs. other tibial fixation) and comparing above primary outcomes.
- Cost-effectiveness analysis.

### Search methods for identification of studies

Electronic and other searches (See Appendix 1 – Supplementary data)

### Data collection and analysis

*Selection of studies:* Both authors screened the titles and abstracts of all studies identified in the search strategy. Full text versions were obtained of all studies identified as being potentially relevant, and were assessed for inclusion, using an eligibility pro forma screening document that was based on pre-specified inclusion/exclusion criteria.

*Data extraction and management:* A data extraction form was developed to aid in the collection of details (data items) from included studies. One review author (JDV) independently extracted the data, and a second review author (MM) validated the extracted data. Appendix 2 – Supplementary data shows this form.

If more than one publication arose from the same study, all versions were considered to maximize data extraction and the primary publication was identified along with the secondary references.

### Assessment of risk of bias in included studies

Studies were assessed using the Cochrane Collaboration tool for assessing risk of bias—at both the study level and the outcome level (Higgins et al. 2008). This tool addresses 6 specific domains, namely sequence generation, allocation concealment, blinding, incomplete outcome data, selective outcome reporting and other issues (e.g. extreme baseline imbalance) (see Appendix 3 – Supplementary data for details of criteria on which the bias judgment was based). Blinding and completeness of outcome data were assessed for each outcome separately. A risk-of-bias table was completed for each eligible study. Any disagreement among the review authors was discussed to achieve a consensus.

An assessment of risk of bias using a “risk of bias summary figure”, which presents all of the judgments in a cross-tabulation of studies by entry was evaluated. This display of internal validity indicates the weight the reader may give the results of each study.

Studies other than RCTs (i.e. quasi-randomized controlled trials) were assessed using the same criteria. We incorporated the results of the risk-of-bias assessment into the review through systematic narrative description and commentary about each of the domains, leading to an overall assessment of the risk of bias of included studies and a judgment about the internal validity of the results.

### Measures of treatment effect

Each study is reported separately. The results of binary outcomes (e.g. revision or not) are presented as risk ratios (RRs) with corresponding 95% confidence intervals (CIs). For continuous data, we used the mean difference if outcomes were measured in the same way between trials. Furthermore, if pooling of data was not possible, we used the statistics used in the study for analysis of treatment effect; in most cases the Mann-Whitney U test was used (a nonparametric test).

### Dealing with missing data

In such cases, we attempted to contact authors where data was missing and requested it. We also addressed the effect of missing data in the discussion section. In the case of abstracts, we attempted to contact authors to determine whether a paper had been published in a peer-reviewed journal. If a paper had been written from an abstract but was unpublished, we attempted to obtain it from the authors.

### Assessment of heterogeneity

Assessment of statistical heterogeneity was done using the I^2^ statistic, in order to determine appropriateness for meta-analysis. If the I^2^ statistic was at or below 60%, the heterogeneity was considered moderate and meta-analysis was considered appropriate. If the value was greater than 60%, sensitivity analysis was undertaken in an attempt to identify which studies were most likely to be causing the problem. If there were only a few such studies, and they could be identified, the reasons for their difference were explored and the appropriateness of removing these studies was determined. When appropriate, meta-analysis excluding any such studies was performed.

### Assessment of reporting biases

We used a funnel plot to assess reporting bias. Each primary outcome was reported separately. Furthermore, an assessment was made of publication bias (including a review of unpublished studies), location bias (types of journals), and language bias.

### Data synthesis

Where possible, we grouped studies together that were similar. In the absence of heterogeneity (I^2^ = 0%) or in the presence of low heterogeneity (I^2^ statistic less than 40%), a fixed-effects model was used. If heterogeneity was moderate (I^2^ statistic greater than 40% and less than or equal to 60%), a random-effects model was used.

## Results

**Figure 1.**
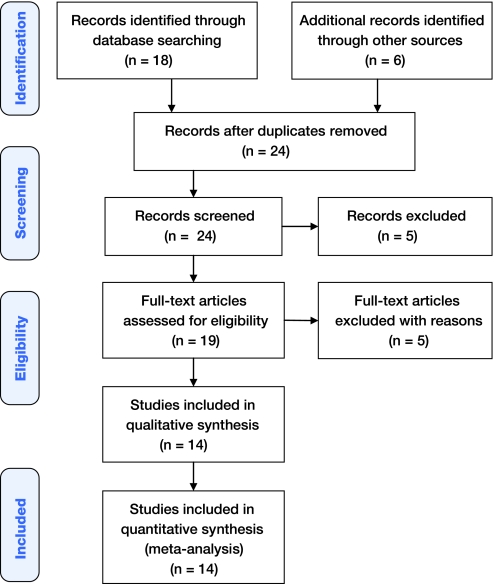
PRISMA 2009 flow diagram used to identify studies for inclusion in HA tibial analysis.

Results of the search: 19 studies were identified for evaluation (see also the PRISMA flow diagram; Figure 1).

### Included studies

We identified 14 prospective randomized trials in which a total knee implant with an HA-coated tibial component was compared to: cemented non-porous coated stemmed metal tibial tray with polyethylene insert bearing surface; porous coated metal-backed tray with polyethylene insert bearing surface (non-cemented); and porous coated metal-backed tray with screw fixation and polyethylene insert bearing surface (non-cemented). No studies were identified that compared an HA-coated tibial component to a cemented all-polyethylene tibial component. This included a total of 926 evaluable total knee implants (Table 1).

**Table 1.** The number of evaluable knees used from each trial and included in outcome analysis

Study	No. of knees	HA group	Other group	Country
Beaupré 2007	81	40	41	Canada
Carlsson 2005	150	50	100	Sweden
Findlay 2007	171	94	77	UK
Hansson 2008	49	24	25	Sweden
Nilsson 1999	57	29	28	Sweden
Nilsson 2006	95	62	33	Sweden
Petersen 2005	16	8	8	Denmark
Pijls 2010	29	9	20	Netherlands
Regnér 1998	40	20	20	Sweden
Regnér 2000	51	25	26	Sweden
Toksvig-Larsen 2000	47	28	19	Sweden
van der Linde 2006	22	12	10	Netherlands
van der Linde 2008	78	38	40	Netherlands
Walker 2000	40	12	28	UK
Total	926	451	475	

Based on the available data, the comparisons evaluated were as follows:

- HA-coated (with or without porous coated tibial undersurface and with or without screw fixation) vs. all other implants (cemented MB and porous-coated MB with or without screw fixation). Evaluation of performance against the RSA (MPTM) endpoint was done at 2 years (Regnér et al. 1998, Nilsson et al. 1999, 2006, Toksvig-Larsen et al. 2000, Carlsson et al. 2005, Hansson et al. 2008, Pijls et al. 2010)
- HA-coated (with or without porous coated tibial undersurface and without screw fixation) vs. other implants (cemented MB and porous coated MB without screw fixation). Evaluation of performance against the RSA (MPTM) endpoint was done at 2 years (Toksvig-Larsen et al. 2000, Nilsson et al. 1999, 2006, Carlsson et al. 2005, Hansson et al. 2008)
- HA-coated (with or without porous coated tibial undersurface and without screw fixation) vs. porous coated MB without screw fixation). Evaluation of performance against the RSA (MPTM) endpoint was done at 2 years (Toksvig-Larsen et al. 2000, Hansson et al. 2008)
- HA-coated (with or without porous coated tibial undersurface and without screw fixation) vs. cemented MB. Evaluation of performance against the RSA (MPTM) endpoint was done at 2 years (Nilsson et al. 1999, 2006, Carlsson et al. 2005)
- HA-coated (with porous coated tibial undersurface and with screw fixation) vs. porous coated MB with screw fixation). Evaluation of performance against the RSA (MPTM) endpoint was done at 2 years (Regnér et al. 1998).
- HA-coated vs. cemented MB tibia – durability (failure requiring a revision) at 5 years (Nilsson et al. 1999, Carlsson et al. 2005, Beaupré et al. 2007)
- HA-coated vs. cemented MB tibia – durability (failure requiring a revision) at 8–10 years (Findlay et al. 2007, Pijls et al. 2010)
- Functional assessment:
  – KSS clinical score at 2 years (Carlsson et al. 2005)—comparing HA separately to cemented and porous or press-fit.
  – HSS score at 2 years (Regnér et al. 1998, van der Linde 2008).
- RAND-36 and WOMAC at 5 years
- HA-coated vs. cemented implants—adverse events (Nilsson et al. 1999, 2006, Carlsson et al. 2005, Beaupré et al. 2007, Findlay et al. 2007, Pijls et al. 2010).

See Appendix 4 – Supplementary data for characteristics of studies included.

### Excluded studies

Two trials were excluded, as they were earlier trials (Önsten et al. 1998, Nelissen et al. 1998) Önsten et al. 1998) was an earlier trial of Carlsson et al. 2005). Note, however, that Önsten et al. 1998) was used for adverse event/complication data, KSS scores, and for randomization scheme. Nelissen et al. 1998) was an earlier trial of Pijls et al. 2010). Note, however, that Nelissen et al. 1998) was used for adverse events, KSS functional scores, and for randomization scheme. One study was excluded (Therbo et al. 2008) due to fact that it did not meet the criteria for inclusion—i.e. it only had one year follow up. One was excluded (Nakama et al. 2006) as it was a protocol only, and 1 was excluded (Uvehammer et al. 2007) because it analyzed the femoral component only. Thus, 5 studies were excluded.

### Risk of bias in included studies

*Allocation.* Sequence generation (randomization scheme used) and allocation to treatment arm were not well defined in the majority of the studies.

*Blinding.* Physicians performing the procedure could not be blinded regarding the treatment arm. In a number of the studies, it was unclear whether patients had also been blinded to the treatment arm. It was also unclear in approximately 50% of the studies whether clinicians evaluating radiographs over time (for potential loosening of the implant) were blinded to the treatment arm. In addition, it was unclear approximately 50% of the time whether clinicians evaluating for functionality, pain, and quality of life were blinded to the treatment arm.

*Incomplete outcome data:* A substantial proportion of the missing data, especially in the longer-term outcome studies, were related to survival of the patient. According to the appendix “risk of bias assessment”, this represents a low risk of bias.

### Overall assessment of reporting bias—funnel plot analysis (not shown)

Durability as measured by RSA with MPTM of > 0.2 mm/2 years constituting instability: Funnel plot analysis of this outcome for the 7 studies included (Regnér et al. 1998, Nilsson et al. 1999, 2006, Toksvig-Larsen et al. 2000, Carlsson et al. 2005, Hansson et al. 2008, Pijls et al. 2010) showed symmetry, indicating that bias was minimal. All other subsets/permutations of this also showed symmetry.

Durability as defined by need for revision at 5 years: Funnel plot analysis of this outcome for the 3 studies included (Nilsson et al. 1999, Carlsson et al. 2005, Beaupré et al. 2007) showed symmetry, indicating that bias was minimal.

Durability as defined by need for revision at 8–10 years: Funnel plot analysis of this outcome for the 2 studies included (Findlay et al. 2007, Pijls et al. 2010) showed symmetry, indicating that bias was minimal.

*Adverse events:* Funnel plot analysis of this outcome for the 6 studies included showed symmetry, indicating that bias was minimal (Nilsson et al. 1999, 2006, Carlsson et al. 2005, Beaupré et al. 2007, Findlay et al. 2007, Pijls et al. 2010).

### Effects of interventions

**Figure 2.**
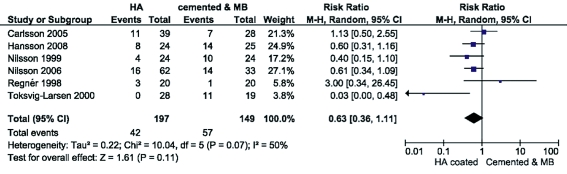
RSA analysis year 2; HA-coated vs. other tibial fixation; all implants.

**Figure 3.**
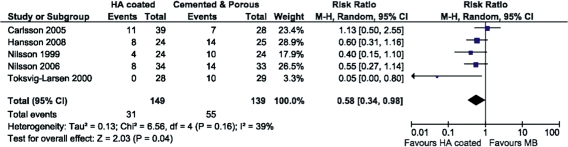
RSA analysis year 2; HA-coated vs. other tibial fixation; all implants except for those with screw fixation of tibial baseplate.

The results of each comparison category are shown in Table 2, Figures 2 and 3). We considered the definition of durability (mainly RSA analysis) to be consistent enough to warrant pooling of data. Secondly, we also accepted that there was variation in the reported definition of adverse events (a second main outcome measure) but we attempted to standardize this definition by not including adverse events that appeared not to result from complications arising from the surgery itself or from the function of the tibial implant. Examples of exclusions included: partial tear of the quadriceps tendon, patellar fracture following a fall, fracture of the femoral neck, and ipsalateral patellar fracture following a car accident. In all of these trials, the evaluation of heterogeneity was low to moderate. Lastly, we realized that there was probably wide variation in the functional assessment based on the various validated measures used, and attempted to pool data where the same validated measure was used.

**Table 2.** Durability (pooled data)

Comparisons made	Trials included	Endpoint	HA pooled sample size	MB pooled sample size	RR (95% CI)	Model used
HA coated (with and without porous coated tibial undersurface) with and without screw fixation vs. MB cemented and porous coated tibial components with and without screw fixation (see Figure 2)	Regnér et al. 1998, Nilsson et al. 1999, Toksvig-Larsen et al. 2000, Carlsson et al. 2005, Nilsson et al. 2006, Hansson et al. 2008, Pijls et al. 2010	MTPM >0.2 mm/2 years	197	147	RR=0.63(0.36–1.11);p=0.11;I^2^ = 50%	M-H random effects
HA coated (with and without porous coated tibial undersurface) without screw fixation vs. MB cemented and porous coated tibial components without screw fixation (see Figure 3)	Nilsson et al. 1999, Toksvig-Larsen et al. 2000, Carlsson et al. 2005, Nilsson et al. 2006, Hansson et al. 2008	MTPM >0.2 mm/2 years	149	139	RR=0.58(0.34–0.98); p=0.04,I^2^ = 39%	M-H random effects
HA coated (with and without porous coated tibial undersurface) without screw fixation vs. MB porous coated components without screw fixation (figure not shown)	Toksvig-Larsen et al. 2000, Hansson et al. 2008	MTPM >0.2 mm/2 years	52	44	RR=0.16 (0.00–5.75);p=0.32;I^2^ = 84%	M-H random effects
HA coated (with and without porous coated tibial undersurface) without screw fixation vs. cemented MB tibial components (figure not shown)	Nilsson et al. 1999, Carlsson et al. 2005, Nilsson et al. 2006	MTPM >0.2 mm/2 years	97	85	RR=0.65(0.41–1.04); p=0.07;I^2^ = 29%	M-H fixed effects
HA coated (with and without porous coated tibial undersurface) vs. cemented MB tibial components (figure not shown)	Nilsson et al. 1999, Carlsson et al. 2005, Beaupré et al. 2007	5 year durability – need for replacement	97	87	RR=1.83(0.34–9.86); p=0.48;I^2^ = 0%	M-H fixed effects
HA coated (with and without porous coated tibial undersurface) vs. cemented MB component (figure not shown)	Findlay et al. 2007, Pijls et al. 2010	8–10 year period – need for replacement	104	88	RR=3.28(0.37–28.7); p=0.28;I^2^ = not applicable	M-H fixed effects

### Other durability data examined—non-pooled

In a separate trial examining the changes in bone mineral density using DEXA analysis between HA-coated (n = 8) and porous coated tibial implants (n = 8), Peterson et al. (2005) found no significant effect of HA coating on bone remodeling in the proximal tibia.

Durability as defined above by Ryd et al. (1995) (MTPM of > 0.2 mm in 2 years for prediction of loosening at 10 years) or abject failure could not be examined in the paper by Walker et al. (2000), as the analyses performed in this trial examined migration in the implant groups only (HA press-fit vs. MB press-fit vs. MB cemented) and did not define failure based on the definition by Ryd et al. (1995). What was found was that migration based on the Walker definition was statistically significantly higher in the MB press-fit group than in the HA press-fit or MB cemented groups when examined for axial migration (p-values not stated). Furthermore, tilt angles about the M-L axis were statistically significantly different between MB cemented and MB press-fit (favoring less migration with MB cemented at 19–30 months postoperatively). There was no statistically significant difference in this parameter between MB press-fit and HA press-fit.

Durability as defined by Ryd et al. (1995) (MTPM of > 0.2 mm in 2 years for prediction of loosening at 10 years) or abject failure could not be examined in the paper by van der Linde et al. (2008) paper, as the analysis performed used the above criterion for prediction of loosening at 10 years. Using this definition, 11 tibial trays from the periapatite (PA) group (26%) and 29 tibial trays from the porous group (63%) could be identified as being at risk of loosening at 10 years. There was no statistical analysis performed with this finding. In addition, in this paper the authors did not identify any implants that required revision.

### Function

Function was evaluated using validated scoring systems as outlined above. Functional scores were reported on below from data at 2 years and 5 years (Table 3).

**Table 3.** Function scores from data at 2 years and 5 years. Statistical method used: Inverse variance, fixed effects analysis model

Comparisons made	Trials included	Endpoint	HA sample size	MB sample size	Differencemean (95% Ci)	p-value
HA coated (porous & press fit) vs. porous MB tibial components	Carlsson et al. 2005	Knee Society Score clinical assessment – 2 year	50	37	1.00 (-3.63 to 5.63)	0.67
HA coated with porous coated tibial undersurface vs. cemented MB tibial components	Carlsson et al. 2005	Knee Society Score clinical assessment – 2 year	50	29	3.00 (-4.35 to 10.35)	0.42
HA coated with porous coated tibial undersurface with screw fixation vs. porous MB tibial components with screw fixation	Regnér et al. 1998	Hospital for Special Surgery (HSS) clinical assessment – 2 year	20	20	-1.00 (-3.60 to 5.66)	0.67
HA coated with porous coated tibial undersurface without screw fixation vs. porous MB tibial components without screw fixation	van der Linde et al. 2008	Hospital for Special Surgery (HSS) clinical assessment – 2 year	38	48	0.00 (-3.86 to 3.86)	1.00
HA coated press fit vs. cemented MB tibial component	Beaupré et al. 2007	RAND-35 at 5 years	40	41	3.46 (-8.23 to 15.15)	0.56
HA coated press fit vs. cemented MB tibial component	Beaupré et al. 2007	WOMAC at 5 years	40	41	-1.40 ( -10.36 to 7.56)	0.76

### Other functional data examined

The functional status (as assessed using the KSS) of Walker et al. (2000) was evaluated based on data presented in the trial, which demonstrated no statistically significant difference between the groups from the KSS score at 2 years (p-values not available) (HA press-fit vs. MB press-fit vs. MB cemented).

### Adverse events

**Figure 4.**
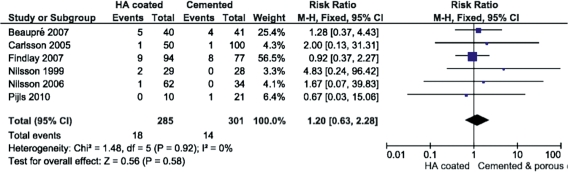
Adverse events; complications resulting from the surgical procedure and/or subsequent need for surgical intervention or immediate medical attention.

Data concerning 586 knees were pooled from 6 trials (Nilsson et al. 1999, 2006, Carlsson et al. 2005, Beaupré et al. 2007, Findlay et al. 2007, Pijls et al. 2010) analyzing adverse events. Adverse events were defined as complications resulting from the surgical procedure and/or subsequent need for surgical intervention or immediate medical attention, and included: DVT, instability due to late varus or valgus with need of surgical intervention, patellofemoral problems with need of surgical intervention, knee flexion problems (including stiffness) with need of surgical manipulation, hematoma requiring evacuation, allergic reactions to medications and infections (over the life of the implant). Adverse events were not significantly different between the groups (RR = 1.20, 95% CI: 0.63–2.28; p = 0.58; I^2^ = 0%; Mantel-Haenszel (M-H) fixed-effects model) (Figure 4).

### Risk of bias across studies

All studies identified had been published in English. However, as noted above in the funnel plot analyses for overall assessment of reporting bias, symmetry was noted, indicating low expectation of reporting bias. Furthermore, in half of the studies identified there were low numbers of patients (≤ 50).

### Additional analysis

All of these studies were conducted outside the US, with 7 of the 14 from Sweden, 1 from Denmark, 3 from the Netherlands, 2 from the UK, and 1 from Canada.

The majority of patients enrolled in the trials were 67–70 years of age. Only 3 trials evaluated patients < 65 years of age (Walker et al. (2000, Nilsson et al. 2006, Beaupré et al. 2007). These studies could not be combined on the durability endpoint due to the fact that different endpoints were analyzed (Beaupré et al. (2007) had a primary endpoint of durability (revision) at 5 years; Nilsson et al. 2006) had a primary endpoint of durability (MPTM > 0.2 mm in 2 years), and Walker et al. (2000) had a primary endpoint of functional status measured with KSS only, and there was insufficient data available).

## Discussion

### Durability

**Figure 5.**
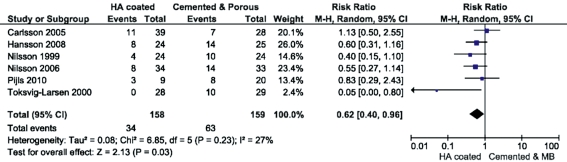
RSA analysis year 2; HA-coated vs. other tibial fixation; all implants except for those with screw fixation of tibial baseplate using an expanded definition of MPTM ≥ 0.2 mm in 2 years.

The results show that there is likely a benefit in reducing the incidence of instability, and hence a need for revision later in the implant life (as measured by MPTM analysis) between the first and second year with HA-coated tibial implants as opposed to all other types of tibial implants when used in patients requiring primary total knee replacement. It should be noted that in the analysis of MPTM, a strict definition of > 0.2 mm in 2 years was used, according to Ryd et al. (1995). However, in one of the trials (Pijls et al. 2010), a definition of “tibial at-risk loosening” of MPTM ≥ 0.2 mm in 2 years was used. If this trial is included in the analysis of HA-coated implants without screw fixation vs. other tibial fixation without screw fixation, the impact of HA coatings on reducing micromotion is strengthened further (RR = 0.62, 95% CI: 0.40–0.96; p = 0.03; I^2^ = 27%; M-H random-effects model) (Figure 5). HA coatings might provide a “biological seal”, preventing the migration of wear debris (i.e. polyethylene, metal) even under unstable conditions (e.g. excessive micromotion) (Rahbek et al. 2000, 2001, 2005).

As mentioned above, when those HA implants with screw fixation were excluded from the analysis (Regnér et al. 1998, Nilsson et al. 2006), a statistically significant result was noted in favor of HA-coated implants. Possible reasons for the instability experienced with the screw-fixated HA tibial components as noted in other studies might be screw osteolysis (Peters et al. 1992, Gejo et al. 2002) from wear debris generated through movement of the tibial insert within the metal-backed tibial component and/or wear debris generated from micromotion at; the screw head/tibial baseplate interface. In any case, screwless fixation of the HA tibial baseplate proved to be superior. The HA coating used in those trials that could be combined for meta-analyses purposes and which proved to be superior (i.e. more stable) was in the thickness range of 30–60 μm with a porous coated pore size in the range of 150–425 μm (Toksvig-Larsen et al. 2000, Carlsson et al. 2005, Nilsson et al. 2006, Hansson et al. 2008). This was in the range of the specifications according to the manufacturer, and was in the thickness range for providing better strength than thicker coatings (Dumbleton et al. 2004). It should also be noted that thicker HA coatings had a tendency to fatigue when placed under load in the laboratory (Dumbleton et al. 2004), and also that in the trial by Regnér et al. 1998), the HA thickness was noted to be in the 80–130 μm range. Possibly the thickness of the HA coating in this trial contributed to instability due to fatigue.

As Ryd et al. (1995) stated above, continuous migration represents defective fixation that manifests very early, and is highly predictive of clinical loosening and revision at year 10. Concerning the outcome of durability as measured by failure at 2 years (requiring revision), there was found to be no difference between HA-coated and cemented MB tibial components. While the trial by Walker et al. (2000) did not use an endpoint that was similar to the others (i.e. MTPM > 0.2 in 2 years), it did demonstrate that the use of HA coating on a press-fit metal-backed implant provided significantly less micromotion than a press-fit metal-backed implant only. This further supports the notion that HA coating alone provides better fixation than implants that are not HA-coated (albeit MB press-fit).

The trial by van der Linde et al. (2008) deviated from the more common endpoint used to determine longer-term durability (i.e. MTPM > 0.2 mm in 2 years). Instead, it used a definition of MTPM > 0.5 mm in 2 years in predicting which implants would be considered loose at 10 years. In using this definition, HA coating added to the porous coated tibial undersurface demonstrated improved fixation compared to a metal-backed porous coated undersurface (p = 0.047). This further supports the notion that HA provides improved fixation over porous coated implants. It is unclear why the authors of this article chose to deviate from the definition by Ryd et al. (1995), and despite repeated attempts to contact the author for an explanation, we did not receive a reply.

### Functionality

As it relates physician- and patient-assessed functioning, there appeared to be no clinically meaningful difference in mean KSS, HSS, WOMAC, and RAND-36 between HA-coated and other tibial implants. One of the issues with the data available was that a pre- and postoperative (2- and 5-year) analysis could not be performed. The only analysis that could be undertaken was to examine a difference between groups regarding function at 2 and 5 years, which again demonstrated no difference.

### Limitations, and other observations

Due to the limited numbers of patients, recommendations on the applicability of the evidence cannot be made with complete confidence. In a prior analysis on small study effects in meta-analysis of osteoarthristis trials (a meta-epidemiological study), it was determined that on average, treatment effects were more beneficial in small trials (< 100) than in large trials (> 100)—leading to a distortion of the results in meta-analyses (Nüesch et al. 2010). It further recommended that the influence of small trials on estimated effects should be routinely assessed. The Cochrane Handbook for Systematic Reviews of Interventions has recommended the following when evaluating treatment effects of small-size studies: “We recommend that when review authors are concerned about the influence of small-study effects on the results of a meta-analysis in which there is evidence of between-study heterogeneity (I^2^ > 0), they compare the fixed- and random-effects estimates of the intervention effect. If the estimates are similar, then any small-study effects have little effect on the intervention effect estimate. If the random-effects estimate is more beneficial, review authors should consider whether it is reasonable to conclude that the intervention was more effective in the smaller studies.” This analysis was performed, and demonstrated similarity in the intervention effect shown in Figure 3 below (random-effects model: RR = 0.58, 95% CI: 0.34–0.98; p=0.04; I^2^ = 39%; vs. fixed-effects model: RR = 0.53, 955, CI: 0.36–0.77; p < 0.001; I^2^ = 39%). Thus, it does appear that HA coating (especially without screw fixation) may promote early fixation/stability compared to other tibial components (cemented and porous coated) in younger patients, as assessed by MPTM at 2 years. The main concern is that there is limited long-term evidence regarding “hard” durability (need for revision/replacement). The functioning of patients with HA coatings appears to be comparable to that with other implant types at 2 and 5 years, regardless of the measure. Adverse events were similar between HA coatings and other implant types.

The trials evaluated in this meta-analysis were performed mainly on patients with osteoarthritis, with a very small minority in patients with rheumatoid arthritis. Nilsson et al. 1999) had 12 patients with RA out of 53; Nilsson et al. 2006) had 23 patients with RA out of 85; and Pijls et al. 2010) had 26 patients with RA and 6 with OA). Unfortunately, none of these patients could be evaluated by outcome as described above to determine whether RA patients with HA-coated implants fared better regarding outcome than those with other types of tibial fixation (as RA patients were not specifically evaluated on the outcomes of durability, functionality, and adverse events).

### Cost-effectiveness analysis

There were no studies that compared the cost effectiveness of HA-coated tibial components with that of any other method of tibial fixation. In the trials that examined patients < 65 years of age, the endpoints varied between studies and further analysis could not be undertaken by combining studies.

**Figure 6.**
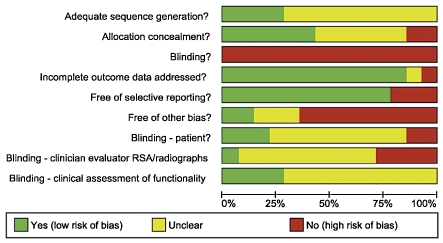
Risk-of-bias graph summary – review of authors' judgments about each risk-of-bias item presented as percentages across all the studies included.

**Figure 7.**
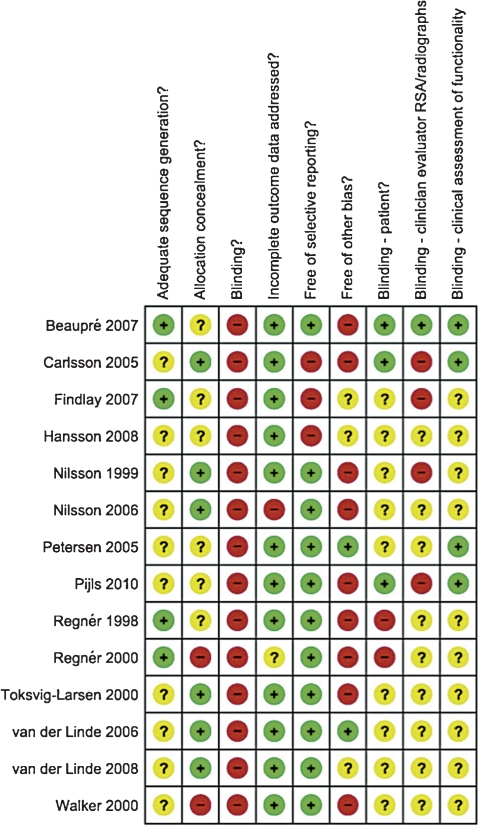
Review of authors' judgments about each risk of bias item for each study included.

The overall quality of the evidence can be considered “average”. It was not possible to blind the clinician performing the procedure. Blinding of patients, of clinicians assessing RSA, and of clinicians evaluating function was not clear in most studies. Also, the randomization method used in allocating patients to treatment groups was not clear in the majority of the studies. Most results (including missing data) were accounted for. Most clinicians performing the trials were funded from outside sources, either industry, medical associations, or foundations. (See Figures 6 and 7 for summary of risk-of-bias assessment).

While no studies were identified in non-English peer-reviewed literature, these studies may exist and were not part of this analysis. However, total joint arthroplasty is practiced mainly in English-speaking countries; thus, is it likely that the majority of studies were identified and analyzed.

### Authors' conclusions

These findings indicate that longer-term durability may be enhanced with HA-coated implants, in patients who are < 70 years of age. Furthermore, it appears that functionality as measured by various validated scoring systems appears to be no different between HA-coated and other tibial implant component types at 2 and 5 years. Thus, in patients who fall within the 65–70 age group, HA coating may be of benefit. Uncemented HA-coated tibial implants may be of use in younger more mobile patients, who may require a second knee implant (revision) later on in life.

Larger-sized studies (i.e. with > 100 patients) should be undertaken to compare the longer-term durability (as measured by need of revision), function, and adverse events with HA-coated implants vs. other porous coated tibial implants in much younger, more active patients (< 65 years of age); durability in patients aged < 65 years (as measured by RSA); and outcome of HA-coated implants in patients with RA.

## Supplementary data

(Appendices) are available at the website (www.actaorthop.org), identification number 4437.
