# Supplementary material for: Hydroxyapatite (HA) coating appears to be of benefit for implant durability of tibial components in primary total knee arthroplasty: A systematic review of the literature and meta-analysis of 14 trials and 926 evaluable total knee arthroplasties
Source: Acta Orthop. 2011 Sep 2;82(4):448–59. doi: 10.3109/17453674.2011.590762 (PMC3237036; doi:10.3109/17453674.2011.590762)
Supplement: Supplementary file 1 [file ORT-1745-3674-82-448-s4437.pdf]

Supplementary article data

Hydroxyapatite (HA) coating appears to be of benefit for implant durability of tibial components in primary total knee arthroplasty

A systematic review of the literature and meta-analysis of 14 trials and 926 evaluable total knee arthroplasties

Jeffrey D Voigt and Michael Mosier

99 Glenwood Rd., Ridgewood, NJ; Washburn University, Topeka, KS, USA
Correspondence JDV: meddevconsultant@aol.com
Submitted 10-10-15. Accepted 11-01-31

Appendix 1: Electronic and other searches

The following electronic searches were conducted:

- The Cochrane Central Register of Controlled Trials (CENTRAL) in the Cochrane Library (Latest issue)
- Ovid MEDLINE (1980 to September 16, 2010)
- Ovid EMBASE (1980 to September 16, 2010)
- EBSCO CINAHL (1982 to September 16, 2010).

A modified search strategy was adopted to search Ovid MEDLINE (with slight modifications for Ovid EMBASE and EBSCO CIHNAHL):

1 exp Arthroplasty, Replacement, Knee/ (8162)
2 knee arthroplasty.tw. (7447)
3 (knee adj3 replace*).tw. (4787)
4 or/1-3 (13029)
5 exp Durapatite/ (8611)
6 hydroxyapatite*.tw. (13565)
7 or/5-6 (16271)
8 4 and 7 (58)
9 randomized controlled trial.pt. (295050)
10 controlled clinical trial.pt. (81966)
11 randomized.ab. (209359)
12 placebo.ab. (123515)
13 clinical trials as topic.sh. (149564)
14 randomly.ab. (154851)
15 trial.ti. (90108)
16 or/9-15 (701541)
17 (animals not (humans and animals)).sh. (3423672)
18 16 not 17 (650507)
19 8 and 18 (19)
20 from 19 keep 1-19 (19)

There were no restrictions on the basis of date or language of publication. This search was performed on June 17, 2010.

A Google search was also executed using the terms: hydroxyapatite, total knee arthroplasty, and randomized controlled trial. The first 15 pages of "hits" were evaluated for identification of prospective randomized trials. This search was performed on September 16, 2010.

Searching of other sources

Corresponding authors of studies that were included and excluded were contacted. The citation lists of papers identified by the above strategies were checked for further reports of eligible studies. Hand searches of the following journals were also undertaken: Journal of Bone and Joint Surgery American (1990 to August 2010); Journal of Bone and Joint Surgery British (1990 to August 2010); Abstract presentations from major orthopedic meetings including the American Academy of Orthopedic Surgery (AAOS); Clinical Orthopedics and Related Research (1990 to August 2010); The Knee (1994 to August 2010); AAOS and the British Orthopaedic Association Clinical Guidelines sections (website); Health Technology Assessment websites including: the Agency for Healthcare Quality and Research (AHRQ); the National Institute for Health and Clinical Excellence (NICE) and the National Institute for Health Research (NIHR) Technology Assessment Programme (UK); the Canadian Agency for Drugs and Technologies in Health (CADTH); the California Technology Assessment Forum (CTAF); BCBS Technology Assessment. Lastly, ongoing prospective randomized clinical trials were searched using the ClinicalTrials.gov website.

Open Access - This article is distributed under the terms of the Creative Commons Attribution Noncommercial License which permits any noncommercial use, distribution, and reproduction in any medium, provided the source is credited.
DOI 10.3109/17453674.2011.590762

Appendix 2: Data extraction form

Data collection form

Name of person/reviewer extracting data:
Author of article:
Title:
Source (e.g. journal title):
Date of study:
Study location (geographical):
Care setting (e.g. hospital):
Inclusion/exclusion criteria (list of patient inclusion and exclusion criteria)
  Inclusion:
  Exclusion:
Sample size:
  number in each arm of trial
  a priori power calculation? (yes/no/not stated)
  trial powered adequately?
Patient baseline characteristics:
  age range:
  gender:
  medical condition(s):

Trial design details

  single center/multicenter trial?
Study type
  randomized controlled trial/matched control/unmatched concurrent control/historic control:
Allocation
  was it random? (yes/no/not stated)
  method of randomization:
  was it concealed? (yes/no/not stated)
Intervention details
  care setting:
  treatment group(s):
  control(s):
  co-interventions:
  duration of intervention:
  who delivered intervention?
  was the provider performing the procedure blinded? (yes/no/not stated)
  was the patient blinded? (yes/no/not stated)
Outcome measures
  what were they?
  methods of assessing outcome measures:
  blind assessment? (yes/no/not stated)
  when were they measured?
  validity of assessment:
  length of follow-up:
Analysis:
  description of analysis employed:
  statistical methods:
  comparisons made:
  intention-to-treat analysis?
  adjustment for confounding?
  subgroups considered:
  exploration of heterogeneity:
Results:
  missing data:
  length of follow up:
  withdrawals/dropouts – are proportion and characteristics of participants lost to follow-up comparable for the study groups at the end of the trial?
  reasons for withdrawal:
  loss to follow-up:
Number of patients with failure on durability (frank failure, pain, loss of functionality requiring revision or with MTPM > 0.2 mm in 2 years (primary outcome):
  intervention arm (1):
  intervention (or control) arm (2):
  intervention arm (if more than 2 intervention arms are included in the trial):
Number of adverse events:
  intervention arm (1):
  intervention (or control) arm (2):
  intervention arm (if more than 2 intervention arms are included in the trial):
Functionality score(s):
  intervention arm (1):
  intervention (or control) arm (2):
  intervention arm (if more than 2 intervention arms are included in the trial):

Conclusions

Implications (e.g. for practice):
Other comments:
  methodological quality of study:
  comparability of intervention:
  baseline comparability:
  funding source(s):
  country of study origin:

Appendix 3: Assessment of risk of bias.

Criteria for a judgment of 'yes' for the sources of bias

1. Was the allocation sequence randomly generated?

Yes, low risk of bias: The investigators describe a random component in the sequence generation process such as: referring to a random number table; using a computer random number generator; coin tossing; shuffling cards or envelopes; throwing dice; drawing of lots.

No, high risk of bias: The investigators describe a non-random component in the sequence generation process. Usually, the description would involve some systematic, non-random

approach—for example: sequence generated by odd or even date of birth; sequence generated by some rule based on date (or day) of admission; sequence generated by some rule based on hospital or clinic record number.

Unclear: Insufficient information about the sequence generation process to permit judgment of “Yes” or “No”.

2. Was the treatment allocation adequately concealed?

Yes, low risk of bias: Participants and investigators enrolling participants could not foresee assignment because one of the following, or an equivalent method, was used to conceal allocation: central allocation (including telephone, web-based, and pharmacy-controlled randomization); sequentially numbered drug containers of identical appearance; sequentially numbered, opaque, sealed envelopes.

No, high risk of bias: Participants or investigators enrolling participants could possibly foresee assignments and thus introduce selection bias, such as allocation based on: using an open random allocation schedule (e.g. a list of random numbers); using assignment envelopes without appropriate safeguards (e.g. if envelopes were unsealed, or non-¬opaque, or not sequentially numbered); alternation or rotation; date of birth; case record number; any other explicitly unconcealed procedure.

Unclear: Insufficient information to permit judgment of “Yes” or “No”. This is usually the case if the method of concealment is not described, or not described in sufficient detail to allow a definite judgment—for example, if the use of assignment envelopes is described, but it remains unclear whether envelopes were sequentially numbered, opaque, and sealed.

3. Blinding: was knowledge of the allocated interventions adequately prevented during the study?

Yes, low risk of bias: Any one of the following:

- No blinding, but the authors of this review judge that the outcome and the outcome measurement were not likely to have been influenced by lack of blinding
- Blinding of participants and key study personnel ensured, and it was unlikely that the blinding could have been broken
- Either participants or some key study personnel were not blinded, but outcome assessment was blinded and the non-blinding of others was unlikely to introduce bias.

No, high risk of bias: Any one of the following:

- No blinding or incomplete blinding, and the outcome or outcome measurement was likely to be influenced by lack of blinding
- Blinding of key study participants and personnel was attempted, but it is likely that the blinding could have been broken
- Either participants or some key study personnel were not blinded, and the non-blinding of others was likely to introduce bias.

Unclear: Any one of the following:

- Insufficient information to permit judgment of “Yes” or “No”
- The study did not address this outcome.

4. Were incomplete outcome data adequately addressed?

Yes, low risk of bias: Any one of the following:

- No missing outcome data
- Reasons for missing outcome data were unlikely to be related to true outcome (for survival data, censoring was unlikely to be introducing bias)
- Missing outcome data balanced in numbers across intervention groups, with similar reasons for missing data across groups
- For dichotomous outcome data, the proportion of missing outcomes compared with observed event risk was not enough to have a clinically relevant effect on the intervention effect estimate
- For continuous outcome data, plausible effect size (difference in means or standardized difference in means) among missing outcomes was not enough to have a clinically relevant effect on observed effect size
- Missing data were imputed using appropriate methods.

No, high risk of bias: Any one of the following:

- Reason for missing outcome data was likely to be related to true outcome, with either imbalance in numbers or reasons for missing data across intervention groups
- For dichotomous outcome data, the proportion of missing outcomes compared to observed event risk was enough to introduce clinically relevant bias in intervention effect estimate
- For continuous outcome data, plausible effect size (difference in means or standardized difference in means) among missing outcomes was enough to induce clinically relevant bias in observed effect size
- “As-treated” analysis done with substantial departure of the intervention received from that assigned at randomization;
- Potentially inappropriate application of simple imputation.

Unclear: Any one of the following:

- Insufficient reporting of attrition/exclusions to permit judgment of “Yes” or “No” (e.g. number randomized not stated, no reasons for missing data provided)
- The study did not address this outcome.

5. Are reports of the study free of suggestion of selective outcome reporting?

Yes, low risk of bias: Any of the following:

- The study protocol is available and all of the study’s pre-specified (primary and secondary) outcomes that are of interest in the review have been reported in the pre-specified way
- The study protocol is not available, but it is clear that the published reports include all expected outcomes, including those that were pre-specified (convincing text of this nature may be uncommon).

No, high risk of bias: Any one of the following:

- Not all of the study’s pre-specified primary outcomes have been reported
- One or more primary outcomes were reported using measurements, analysis methods, or subsets of the data (e.g.

subscales) that were not pre-specified

- One or more reported primary outcomes were not pre-specified (unless clear justification for their reporting was provided, such as an unexpected adverse effect)
- One or more outcomes of interest in the review were reported incompletely so that they cannot be entered in a meta-analysis
- The study report failed to include results for a key outcome that would be expected to have been reported for such a study.

Unclear: Insufficient information to permit judgment of "Yes" or "No". It is likely that the majority of studies will fall into this category.

6. Other sources of potential bias:

Yes, low risk of bias: The study appears to be free of other sources of bias.

No, high risk of bias: There is at least one important risk of bias. For example, the study:

- Had a potential source of bias related to the specific study design used; or
- Stopped early due to some data-dependent process (including a formal-stopping rule); or
- Had extreme baseline imbalance; or
- Has been claimed to have been fraudulent; or
- Had some other problem.

Unclear: There may be a risk of bias, but there is either: insufficient information to assess whether an important risk of bias exists, or insufficient rationale or evidence that an identified problem will introduce bias.

Appendix 4: Characteristics of studies included

Beaupré 2007

Methods

RCT; patients randomized either to cementless tibial fixation with HA or to cemented tibial fixation. Randomization sequence computer generated in blocks of 20 subjects, and randomization codes were stored in sequentially numbered opaque envelopes. The Scorpio Series-7000 standard tibial tray with and without HA (Stryker Orthopedics, Mahwah, NJ) was used; multicenter (3) trial. Implant configuration/shape was identical between the 2 tibial components. The undersurface of the tibial component for both uncemented and cemented tibial designs (interfacing with bone) was bead blasted satin finish in a waffle pattern. Approved by hospital IRBs.

Blinding: no to clinician performing procedure; yes to physiotherapist performing follow-up functional assessment; patients receiving implants were probably blinded to type of tibial implant, as both implants looked exactly the same on radiographic examination.

Intention-to-treat analysis: yes.

A priori power calculation: yes, powered to detect a 10-point difference between groups on either the functional or pain scale of WOMAC ($\alpha = 0.05$, $\beta = 0.20$, power = 80%).

Reliable primary outcomes: yes.

Participants

Male and female. Mean age in HA group: 63.9 (5.8); 15 males and 25 females; mean age in MB cemented group: 62.9 (6.4); 16 males and 25 females. Patients undergoing total knee arthroplasty for a primary diagnosis of non-inflammatory osteoarthritis. Approved by ethics committee at hospital and informed consent obtained.

Exclusion criteria: history of knee infection; previous patellectomy or high tibial osteotomy; deficient posterior cruciate ligament; knee flexion contracture or a varus-valgus deformity of > 20°; < 90° of knee flexion; tibial or femoral bone deficiency that would require augmentation, or > 70 years of age.

Total number of patients randomized to trial: 81.

Interventions

HA group: (n = 40) – uncemented metal-backed porous and HA-coated stemmed metal tray with snap-in polyethylene insert.

MB cemented group (n = 41) – completely cemented metal-backed tray with snap-in polyethylene insert.

Outcomes

Durability of tibial fixation as measured by the revision rate at 5 years.

Functionality measured using the WOMAC functional and pain scale (patient-reported outcomes). RAND-36 also used to determine overall health status. Assessments made at 6 months, 1 year, and 5 years.

Adverse events: intraoperative and postoperative complications including the need for additional surgery.

Notes

Disclosures: One or more of the authors had outside funding or grants in excess of $10,000 from Stryker Canada.

Country of origin: Canada

Most patients did not have patella resurfaced.

Of 81 subjects enrolled, 70 completed 5-year assessment: one died in hospital; and 10 subjects withdrew from trial for various reasons. 6-month scores had to be imputed for 8 subjects whereas 1 scores had to be imputed for 3 patients.

Adverse events included: 3 patients in each group had postoperative wound redness or cellulitis, which resolved with oral administration of antibiotics. Three subjects (one in cemented group and 2 in HA group) required manipulations within the first 6 months postoperatively due to poor knee flexion. One patient in HA group required revision to thicker poly tibial liner 3 years after surgery; and one patient in cement group required a patellar revision two years after surgery.

Carlsson 2005

Methods

RCT; patients randomized in 2 series:

Series #1: randomized to cemented tibia fixation (C-F), uncemented tibial fixation (UC-F) (porous coated titanium beads), or uncemented with HA tibial fixation (porous coated titanium beads and HA coating) (UCHA-F). The Press-Fit Condylar (PFC) modular, posterior cruciate retaining prosthesis with a posterior lipped poly insert was used in all cases (J&J, New Milton UK). Approved by hospital IRB, Lund University.

Series #2: Bilateral knees in all cases; first knee randomly assigned to: C-F, UC-F, or UCHA-F. Second knee implanted with 2 remaining types of fixation also randomized. Same PFC implant used.

Three series of 50 envelopes were randomized into 2 numbered sequences, one for series #1 and one for series #2.

Blinding: no to clinician performing procedure; no to radiographers evaluating patient's tibial fixation; yes to patients receiving implants.

Intention-to-treat analysis: no.

A priori power calculation: no.

Reliable primary outcomes: no.

Participants

Series #1: Male and female. Mean age in cemented fixation group: 72 (7); 7 males and 22 females; mean age in uncemented group: 74(6); 7 males and 27 females; Mean age in uncemented HA group: 72(5); 8 males and 22 females. Patients undergoing total knee arthroplasty for a primary diagnosis of non-inflammatory osteoarthritis. Approved by ethics committee at hospital and informed consent obtained.

Exclusion criteria: rheumatoid arthritis; revision; history of joint sepsis; recent systemic cortiocosteroids, primary or secondary carcinoma in last 5 years; metabolic bone disease such as Paget's disease; psychological disorders limiting rehab; previous intraarticular fracture; varus-valgus deformity of > 20°; < 90° of knee flexion; tibial or femoral bone deficiency that would require augmentation; unsuitable for cruciate retaining arthroplasty; unsuitable for cementless fixation of tibia; previous proximal tibial osteotomy; > 80 years old

Total number of patients/knees randomized to series #1: 88/100.

Series #2: Male and female. Mean age in group: 73 (4); 6 males and 24 females.

Exclusion criteria: revision; history of joint sepsis; recent systemic cortiocosteroids; primary or secondary carcinoma in last 5 years; metabolic bone disease such as Paget's disease; psychological disorders limiting rehab; previous intraarticular fracture; varus-valgus deformity of > 20°; < 90° of knee flexion; tibial or femoral bone deficiency that would require augmentation; unsuitable for cruciate retaining arthroplasty; unsuitable for cementless fixation of tibia; previous proximal tibial osteotomy; > 80 years old

Total number of patients/knees randomized in series #2: 30/60.

Interventions

Series #1: Unilateral.

C-F group: (n = 29) – cemented metal-backed non-porous coated stemmed metal tray with snap-in polyethylene insert.

UC-F group (n = 41) – uncemented metal-backed porous coated tray with snap-in polyethylene insert.

UCHA-F group (n = 30) – uncemented metal-backed porous coated tray with HA coating and snap-in polyethylene insert.

Series #2: Bilateral – 30 patients in total.

C-F/UC-F group: (n = 10) – cemented metal-backed non-porous coated stemmed metal tray with snap-in polyethylene insert in one knee and uncemented metal-backed porous coated implant in other knee.

C-F/UCHA-F group (n = 10) – cemented metal-backed non-porous coated tray with snap-in polyethylene insert in one knee and uncemented metal-backed porous coated tray with HA coating and snap-in polyethylene insert in other knee.

UC-F/UCHA-F group (n = 10) – uncemented metal-backed porous coated tray and snap-in polyethylene insert in one knee and uncemented metal-backed porous coated tray with HA coating and snap-in polyethylene insert in other knee.

Outcomes

Durability of tibial fixation as measured by the revision rate at 5 years and by radiostereometric analysis (RSA) and MTPM.

Functionality measured using the KSS clinical rating system. Assessments made at 3, 12, and 24 months.

Adverse events: intraoperative and postoperative complications including the need for additional surgery.

Notes

Disclosures: No direct funding from outside source; however, benefits were received and were directed to research fund, educational funds, or other non-profit.

Country of origin: Sweden.

4 randomization errors occurred in series #1: in 2 cases, patients who should have been excluded were allocated to trial; in one patient, the wrong implant was used; and in another the correct size of the implant was not available to randomize the patient.

The majority of patients/knees did not receive a patellar implant – only 3 of 116 knees.

One infection noted in porous implant group (patient excluded from analysis); one reoperation in porous group for insertion of a patella component; one reoperation for replacement of a tibial insert in HA group (excluded); one skin necrosis in HA group over tibial tuberosity that required a split-thickness skin graft soon after arthroplasty; one patient was excluded due to insufficient number of tantalum markers (excluded); 3 patients in porous group were excluded for failing to show up at 24-month follow-up.

Findlay 2007

Methods

RCT; patients randomized either to cemented AGC TKA or to HA-coated TKA (undersurface plasma sprayed, porous coating of titanium (thickness 635–888 µm, porosity 35%, pore size of 90–180 µm)) using a random number table to determine the choice of implant. The cemented and HA-coated posterior cruciate retaining TKR was used (Biomet, Warsaw, IN). Ethical approval granted by Conquest Research and ethics committee. Patients signed informed consent forms.

Intent to treat analysis: no.

A priori power calculation: not stated in paper.

Reliable primary outcomes: yes.

Participants

Patient characteristics: 112 females/98 males; mean age 67.5 (47–75 years); all with OA.

Exclusion criteria: > 75 years of age; rheumatoid arthritis.

Number of patients/knees randomized to trial: 210/254.

Interventions

HA group: 136 knees; of these, 31 patients died and 11 were lost to follow-up. This left a total of 94 knees to evaluate on endpoints.

*C*emented group: 118; of these, 27 died and 14 were lost to follow-up. This left a total of 77 knees to evaluate on endpoints.

Outcomes

Durability: implant survival (need for revision).

HSS scores: preoperatively, 6 weeks postoperatively, 6 months, 1 year, and annually thereafter.

Adverse events/complications reported.

Notes

Disclosures: not noted.

Country of origin: England.

Mean follow-up was 8.1 years with a range of 6–13 years.

Revisions: HA group: 2 for tibial aseptic loosening (tibial migration) – due to malalignment and undersizing of tibial component; 2 for patella revisions (according to e-mail from Dr Findlay on July 22, 2010); cemented: none.

Adverse events/complications: HA group: 1 infection; 8 manipulations under anesthetic. Cemented group: 1 infection; 6 manipulations under anesthetic, 1 patella dislocation (according to e-mail from Dr Findlay on July 22, 2010 – dislocation reduced in the Accident and Emergency Deptartment).

As it relates to HSS scores (according to e-mail from Dr Findlay on July 22, 2010), the raw data for the standard deviations was not available – thus, these data could not be included in the HSS analysis for 2-year HSS scores.

Hansson 2008

Methods

RCT; patients randomized either to porous and HA- (Peri-Apatite) coated tibial and HA-coated femoral implant or to porous coated tibial and femoral implant A (non-HA for both arms). Randomization by closed envelope. The Duracon modular knee was used in both cases. Approved by IRB. All patients diagnosed with gonarthrosis (osteoarthritis).

Intention-to-treat analysis: no.

A priori power calculation: yes, migration of 0.15 mm (83% power to find a significant ($p < 0.05$) decrease in migration in 2 groups of 30 patients.

Reliable primary outcomes: yes.

Participants

Unclear as to patient characteristics included in trial.

Exclusion criteria: unclear.

Total number of patients randomized to trial: 60.

Interventions

HA group: (n = 32) – uncemented MB porous coated stemmed metal tray with HA coating and with snap-in polyethylene insert.

MB porous coated group (n = 28) – uncemented MB porous coated stemmed metal tray with snap-in polyethylene insert.

Outcomes

Durability of tibial fixation as measured by radiostereometric analysis (RSA) and MTPM at day 2–4; week 6; and 3, 6, 12, and 24 months.

Functionality measured using the HSS clinical rating system. Assessments made at: 6 weeks, 3, 6, 12, and 24 months.

Notes

Disclosures: not noted.

Country of origin: Sweden.

Adverse events were not reported in trial.

Patella was not resurfaced.

Nilsson 1999

Methods

RCT; patients randomized either to HA-coated tibial fixation or to cemented tibial component. Allocation made to a treatment group by opening a sealed envelope. All tibial components were Tricon II stemmed tibial components (Smith & Nephew, Memphis, TN). Unclear if informed consent was obtained. Tricon II tibial component (titanium alloy) undersurface was grit-blasted with large grooves and a trapezoidal polished central stem (non-porous coated).

Blinding: double blinded – patients blinded to treatment arm; clinician assessing function of implant was also blinded;

radiographer was not blinded and clinician who implanted prosthesis was not blinded.

Intention-to-treat analysis: no.

A priori power calculation: no.

Reliable primary outcomes: no.

Participants

Mean age (range) in HA group (OA/RA): 71 (63–80)/53 (44–64); numbers of OA/RA patients: 20/7; 8 males and 19 females.

Mean age in MB cemented group (OA/RA): 73 (60–83)/71 (61–74); numbers of OA/RA patients: 21/5; 11 males and 15 females. Patients undergoing total knee arthroplasty. Approved by ethics committee at hospital and informed consent was obtained.

Exclusion criteria: unclear.

Total number of patients/knees randomized in trial: 53/57.

Interventions

HA group: (n = 29 knees) – uncemented metal-backed porous coated stemmed metal tray with HA coating and with snap-in polyethylene insert.

*C*emented group (n = 28 knees) – cemented metal-backed non-porous coated stemmed metal tray.

Outcomes

Durability of tibial fixation as measured by radiostereometric analysis (RSA) and MTPM at week 1 (reference exam); at week 6; at 3, 6, and 12 months; and at 2 and 5 years.

Functionality was measured using the KSS clinical rating system. Assessments were made at 6 weeks, 2 years, and 5 years.

Adverse events: intraoperative and postoperative complications including the need for additional surgery.

Notes

Disclosures: Supported by grants from the Swedish Medical Research Council.

Country of origin: Sweden.

Thirteen patients (14 knees) lost for various reasons during the 5-year follow-up: 3 patients were revised, 8 patients died of unrelated causes, and 1 patient sustained a cerebral infarction 4 years postoperatively.

Complications included: HA group – 1 patient: revision 7 months postoperatively due to loosening of tibial component (most likely due to carrying his ill wife 8 weeks after receiving implant); 2 patients: revised between years 1 and 2 due to suspected infection; 1 patient: ipsilateral supracondylar femoral fracture after severe fall between years 2 and 4 postoperatively. Cemented group – 2 patients: ipsilateral supracondylar femoral fracture after severe fall between years 2 and 4 postoperatively; 1 patient: aseptic necrosis of the patella 6 months postoperatively (patient treated conservatively).

All patients received a cemented all-poly patellar component.

Nilsson 2006

Methods

RCT; patients randomized to either cemented metal-backed non-porous tibial component; uncemented (HA-coated) with porous coated tibial component with screws; or uncemented (HA-coated) with porous coated tibial component without screws. Randomization between the 3 modes of tibial fixation was accomplished by opening a sealed envelope during the operation. Profix TKA system (Smith & Nephew, Memphis, TN) was used – different stem designs between cemented and uncemented groups. Study approved by IRB.

Blinding: no regarding clinician performing procedure; unclear regarding others.

Intention-to-treat analysis: no.

A priori power calculation: no.

Reliable primary outcomes: yes.

Participants

HA group with screws: M/F % = 43/57; median age = 56.5 (29–63); OA:RA = 22:6; patellar implant (yes:no) = 9:19.

HA group without screws: M/F % = 35/65; median age = 56 (39–64); OA:RA = 28:7; patellar implant (yes:no) = 11:24.

Cemented MB group: M/F % = 41/59; median age = 55.5 (34–64); OA:RA = 24:10; patellar implant (yes:no) = 5:29.

Exclusion criteria: > 65 years; > 150 kg; previous infection; malignant disease; severe osteoporosis.

Total number of patients/knees randomized: 85/97.

Interventions

HA with screws (n = 28 knees).

HA without screws (n = 35 knees).

MB cemented fixation (n = 34 knees).

Outcomes

Durability of tibial fixation as measured by radiostereometric analysis (RSA) and MTPM at 6 weeks; 3, 6, 12, and 24 months.

Functionality measured using the KSS and HSS clinical rating system. Assessments made at: 6 weeks; 3, 6, 12, and 24 months.

Adverse events: intraoperative and postoperative complications including the need for additional surgery.

Notes

Disclosures: One or more authors received funding from Smith & Nephew and Umeå University, Sweden.

Country of origin: Sweden.

Complications included: one transient partial peroneal nerve palsy in cemented group that resolved completely in 3 months (no intervention). (Note: this was not counted as an adverse event due to definition used above); one patient in HA group (with screw fixation) – patella component inserted after one year due to symptomatic patellofemoral arthrosis (according

to follow-up e-mail from Dr Nilsson on July 13, 2010 – saved as PDF file).

According to e-mail from Dr Nilsson on July 12, 2010, 14 cemented knees were classified as unstable using MPTM definition, 8 HA with screw fixation that were unstable, and 8 HA with no screw fixation that were unstable. E-mail response saved as PDF file.

Petersen 2005

Methods

RCT; patients randomized either to uncemented tibial fixation with HA coating on wire mesh or to uncemented tibial fixation with wire mesh only. Unclear as to randomization method/scheme used. All prostheses were posterior cruciate retaining total condylar implants (Interax; Howmedica, Rutherford, NJ). Approved by IRB and informed consent obtained. Wire mesh had window in the mesh for osseous ingrowth of 2.25 mm^2.

Blinding: no regarding clinician performing procedure; unclear regarding others.

Intention-to-treat analysis: no.

A priori power calculation: no.

Reliable primary outcomes: yes.

Participants

HA and porous coated tibia: 8 women with mean age 70 (68–76) years.

Porous coated tibia: 6 women and 2 men, mean age 77 (72–82) years.

Interventions

HA and porous coated tibia (n = 8).

Porous coated tibia (n = 8).

Total patients enrolled: 16, not including the 2 explained exclusions below.

Outcomes

Durability of tibial fixation as measured by DEXA at 2 weeks; 3, 6, 12, and 24 months.

Notes

Disclosures: No benefits or funds were received for this study.

Country of origin: Denmark.

No adverse events reported on.

One patient died within three months of procedure and one patient was excluded due to postoperative psychosis.

Pijls 2010

Methods

RCT; patients randomized to cemented tibial fixation (with smooth diamond tibial undersurface), uncemented tibial fixation (with mesh-wire tibial undersurface; non-porous coated), or HA-coated tibial fixation (with HA plasma sprayed on to mesh-wire tibial undersurface; non-porous coated). Allocation made to treatment group by a random number. All prostheses were posterior cruciate retaining total condylar implants (Interax; Howmedica, Rutherford, NJ). Approved by IRB. Wire mesh had window in the mesh for osseous ingrowth of 2.25 mm^2.

Blinding: double blinded – patient blinded to treatment arm; clinician assessing function of implant also blinded; radiographer was not blinded and clinician who implanted prosthesis was not blinded. [OK?]

Intention–to-treat analysis: no.

A priori power calculation: no.

Reliable primary outcomes: yes.

Participants

No difference between groups: average age: 68 (11.6) years (p = 0.1); BMI: 23 (2.8) kg/m^2; OA:RA = 5/26.

Exclusion criteria: unclear.

Total number of knees randomized: 31.

Interventions

HA group: (n = 10) – uncemented MB porous coated stemmed metal tray with HA coating and with snap-in polyethylene insert.

MB porous coated group (n = 10) – uncemented MB porous coated stemmed metal tray with snap-in polyethylene insert.

Cemented MB group (n = 11) – cemented MB non-porous coated stemmed metal tray.

Outcomes

Durability of tibial fixation as measured by radiostereometric analysis (RSA) and MTPM at day 2–4; week 6; and 3, 6, and 12 months; and 10 years. After year 1, standard radiographs were used. Functionality measured using the KSS clinical rating system. Assessments made at: 3 and 6 weeks, and 3, 6, and 12 months.

Adverse events: intraoperative and postoperative complications including the need for additional surgery.

Notes

Disclosures: No direct funding from outside source; however, benefits were received and were directed to research fund, educational funds, or other non-profit.

Country of origin: the Netherlands.

One infection developed 6 months postoperatively in a component fixed with cement.

In this trial, a MPTM of ≥ 0.2 mm in 2 years was used, and

not the strict Ryd (1995) definition of MPTM > 0.2 mm in 2 years.

Regnér 1998

Methods

RCT; patients randomized either to uncemented tibial fixation with HA and porous coating or to uncemented tibial fixation with porous coating only. Minimization method of randomization used for treatment allocation. All prostheses were Miller-Galante II. The tibial component was also fixated with 4 screws. Approved by IRB; informed consent was obtained.

Blinding: no regarding clinician performing procedure; no regarding patient, due to randomization method used.

Intention–to-treat analysis: no.

A priori power calculation:

Reliable primary outcomes: yes.

Participants

Unclear regarding patient demographics within each group. As an aggregate, there were 18 men and 18 women with a mean age of 67 (57–77). Allocation (minimization method) based age (< 65 vs. ≥ 65 years), weight (< 75 kg vs. ≥ 75 kg), presence or absence of smoking; gender, degree of deformity (< 10° vs. ≥ 10° varus or valgus deformity).

Total number of patients/knees enrolled: 36/40.

Interventions

HA porous coated tibia (n = 20 knees).

Porous coated tibia (n = 20 knees).

Outcomes

Durability of tibial fixation as measured by radiostereometric analysis (RSA) and MTPM at 1 week and 2, 12, and 24 months.

Functionality measured using the HSS clinical rating system. Assessments made preoperatively and at 2 years.

Notes

Disclosures: Supported by various foundations and medical societies.

Country of origin: Sweden.

All patients received an all-plastic and cemented patellar component.

Did not report on adverse events/complications.

Regnér 2000

Methods

RCT; patients randomized either to uncemented tibial fixation with HA coating or to uncemented tibial fixation with porous coating only. Minimization method of randomization used for treatment allocation. HA-coated prosthesis used was Freeman-Samuelson (FS HA without porous coated tibial undersurface (smooth metal undersurface coated with HA)) and porous coated Miller-Galante II (MG II) prosthesis. All tibial components were also fixated with 4 screws. Approved by IRB; informed consent was obtained.

Blinding: no regarding clinician performing procedure; no regarding patient receiving implant.

Intention-to-treat analysis: no.

A priori power calculation: no.

Reliable primary outcomes: yes.

Participants

17 men and 28 women with a mean age of 67 (56–73) years. Allocation (minimization method) based on age (< 65 vs. ≥ 65 years), weight (< 75 kg vs. ≥ 75 kg), presence or absence of smoking; gender; degree of deformity (< 10° vs. ≥ 10°varus or valgus deformity).

Interventions

FS HA: 25 knees.

MG II: 26 knees.

Total number of patients/knees: 45/51.

Outcomes

Durability of tibial fixation as measured by radiostereometric analysis (RSA) and MTPM at 1 week and 2, 6, 12, 24, 36, and 60 months. Standard radiographs also performed at 1,3, and 5 years postoperatively.

Functionality measured using the HSS clinical rating system. Assessment was made at 5 years only.

Adverse events/complications reported.

Notes

Disclosures: Funds for support of study received from Protek and Zimmer and as well from medical associations and foundations.

Country of origin: Sweden.

One MG II was infected before 1-year follow–up, and was revised. Two patients with FS HA prostheses died 35 and 53 months postoperatively unrelated to surgery.

Toksvig-Larsen 2000

Methods

RCT; unclear regarding randomization scheme; unclear if patient consent or IRB approval. Groups 1–3 below used the Osteonics 7000 total knee system; group 4 used the Howmedica Duracon system.

Blinding: no regarding clinician performing procedure; unclear regarding others.

Intention-to-treat analysis: no.

A priori power calculation: no.

Reliable primary outcomes: yes.

Participants

15 men and 45 women; mean age 71 (44–84) years; mean weight 78 (49–125) kg.

Total number of patients/knees: 60/62.

Interventions

Group 1 (n = 15 patients): porous coated Osteonics tibial tray using internally cooled oscillating saw blade (Cool Cut, Mitab, Sweden).

Group 2 (n = 15 patients): porous coated Osteonics tibial tray using standard oscillating saw blade (3M Maxi Driver L122 oscillating sawblade; 3M, St. Paul, MN).

Group 3 (n = 16 patients): waffled CoCr surface (non-porous coated) (flat surface) Osteonics tibial tray with HA coating using internally cooled oscillating saw blade (Cool Cut, Mitab, Sweden).

Group 4 (n = 16 patients): porous coated CoCr Duracon (Howmedica) cruciform tibial tray with HA coating using internally cooled oscillating saw blade (Cool Cut, Mitab, Sweden).

Outcomes

Durability of tibial fixation as measured by radiostereometric analysis (RSA) and MTPM immediately postoperatively; 6 weeks, 6 months, 1 year, and 2 years.

Functionality measured using the HSS clinical rating system. Assessments were made preoperatively, year 1 and year 2.

Notes

Disclosures: Funding from Howmedica, Lund University Medical Faculty, and research council.

Country of origin: Sweden.

Only 8 patellas were resurfaced with a cemented all-poly tibial component: group 1 (2); group 2 (3); group 3 (1); and group 4 (2).

Adverse events not reported.

One implant (porous coated without HA coating; unclear whether group 1 or 2) was revised after 2 years due to severe subsidence, increasing varus alignment, and major loss of bone stock.

No benefit from using saline-cooled saw blade.

van der Linde 2006

Methods

RCT; unclear regarding randomization scheme. Patients randomized during procedure. IRB approval (including informed consent). Use of Duracon TKA – HA (with metal porous coated tibial undersurface) and porous coated tibial implant, posterior stabilized (Stryker, Montreux, Switzerland).

Blinding: no regarding clinician performing procedure; unclear regarding others.

Intention-to-treat analysis: no.

A priori power calculation: yes – suggesting that a significant difference in subsidence of 0.3 mm could be observed when 12 knees were included in each RA group.

Reliable primary outcomes: yes.

Participants

HA-coated with porous coated tibial undersurface group: F/M = 10/2; age = 65 (8.3) years; BMI = 25 (4.3).

Porous group: F/M = 8/2; age = 62 (2) years; BMI = 26 (2.9).

All with rheumatoid arthritis.

Total number of patients/knees: 21/26.

Interventions

HA (n = 12).

Porous coated (n = 10).

Outcomes

Durability as measured at year 2: y-plane subsidence of tibial component.

Functionality: HSS preoperatively and at 24 months.

Notes

Disclosures: no commercial interests.

Country of origin: the Netherlands.

Adverse events/complications: none mentioned in study.

One patient died unrelated to knee prosthesis procedure before 1-year follow-up.

van der Linde 2008

Methods

RCT; unclear regarding randomization scheme. Patients randomized during procedure. IRB approval (including informed consent). Use of Duracon TKA – PA (Periapatite) with porous coated tibial undersurface (porous coated multiple layer bead ingrowth surface) and porous coated tibial implant, posterior cruciate retaining, non-constrained system (Stryker Howmedica Osteonics)

Blinding: no regarding clinician performing procedure; unclear regarding others.

Intention-to-treat analysis: no.

A priori power calculation: no.

Reliable primary outcomes: yes.

Participants

PA-coated with porous coated undersurface group: F/M = 33/10; age = 71 (7.8) years; BMI = 29 (4.7).

Porous group: F/M = 31/12; age = 71 (7.27) years; BMI = 30 (4.9).

Total knees/patients: 86/86 entered into trial.

Interventions

PA (n = 43).

Uncoated (n = 43).

Outcomes

Durability of tibial fixation as measured by RSA with (1) a translation rate during the second postoperative year of MTPM > 0.5 mm in 2 years along one or more coordinate axes (Ryd 1995) and/or (2) rotation > 1° about one or more coordinate axes, as defining aseptic loosening – with patients evaluated preoperatively, one week before mobilization, 3 months, 6 months, 1 year, and 2 years postoperatively.

Insall (HSS) score used for clinical assessment.

Notes

Disclosures: No disclosures noted.

Country of origin: the Netherlands.

Adverse events/complications: none noted in study.

Of a total of 86 patients entered into the trial, 5 PA-coated and 3 uncoated were lost to follow-up. In the PA group, 3 patients died after 1 year of follow-up, one patient had a CV accident within 2 weeks of operation, and a fifth patient moved abroad. In the uncoated group, one patient died and one moved after one year of follow-up, and a third patient underwent an upper arm amputation in another hospital due to vascular problems; RSA follow-up was deemed too short to define stability. In total, there were 38 evaluable PA patients and 40 uncoated patients.

We sent a follow-up e-mail inquiry to E. Valstar on August 31, 2010 to determine why a different definition of aseptic loosening was used in this trial, and why the Ryd (1995) definition was not used. (The definition of durability in this paper was different from the Ryd (1995) paper. While the Ryd paper used the predictor of loosening at 10 years of MTPM > 0.2 mm in 2 years, this paper used a definition of MTPM > 0.5 mm in 2 years). Dr. Valstar in turn forwarded this to Eric Garling at Stryker, the main author of the paper, who used this in his PhD thesis. We re-sent the inquiry to Eric Garling on September 12, 2010 requesting additional clarifications on the article. These inquiries were never responded to by E. Garling.

Walker 2000

Methods

RCT; Patients were randomized just prior to procedure occurring. Unclear regarding randomization scheme, but patients were allocated by a card being removed from an envelope. Study received IRB approval. Use of Kinemax Condylar TKA – HA-coated press fit (non-porous coated), MB press fit (non-porous coated implant) and MB cemented implant (Howmedica Ltd., Pfizer Hospital Products).

Blinding: no regarding clinician performing procedure; unclear regarding others.

Intention-to-treat analysis: no.

A priori power calculation: no.

Reliable primary outcomes: yes.

Participants

HA-coated press fit group: F/M = 10/2; mean age = 61.3 years; OA/RA = 54.5/45.5.

Non-cemented press-fit: F/M = 9/5; mean age = 64.7 years; OA/RA Dx = 71/6.4.

Cemented: F/M = 12/2; mean age = 64.4 years; OA/RA Dx = 50/37.6.

Total number of patients/knees = 40/40.

Exclusion criteria: history of infection, poor bone stock, poor physical condition, and steroid therapy.

Interventions

HA (n = 12).

Non-cemented press-fit (n = 14).

Cemented MB (n = 14).

Outcomes

Migration as measured at year 2 via axial migration (transverse or varus-valgus); varus-valgus rotations; and tilt angles about the M-L axis. However, there was no analysis of potential for loosening as per Ryd (1995).

Clinical results (including pain assessment): use of Knee Society Score preoperatively, at discharge, at 6 months, 1 year, and 2 years.

Notes

Performed in the UK by 5 clinicians at 4 different centers.

Disclosures: study funded by Howmedica Ltd., Pfizer Hospital Products.

Adverse events were not reported.

The analysis of migration used in this study (while 3-dimensional and validated in extensive laboratory experiments (according to e-mail from author sent on September 7, 2010) – A/P; M/L, and varus-valgus tile) was different from Ryd (1995), which used 3-dimensional analysis to determine MTPM of > 0.2 mm in 2 years. (MTPM is a 3-dimensional vector (the x-plane corresponds to medial migration of component, the y-plane corresponds to proximal migration, and the z-plane corresponds to posterior migration).

We sent an e-mail to P.S. Walker on September 5, 2010 asking if there was any sort of analysis undertaken that examined the migration seen with each tibial fixation method used and prostheses at risk of loosening at 10 years (according to Ryd (1995) – i.e. MTPM > 0.2 mm between year 1 and year 2 is highly predictive of loosening at year 10 (with a predictive power of 85%)). On September 8, 2010, the author –replied by e-mail that an analysis of the prediction of loosening had not been undertaken.
